# An Atypical Presentation of Disseminated Intravascular Coagulation Following Ocular Interventions for Proliferative Diabetic Retinopathy: Mimicking Retrobulbar Hemorrhage

**DOI:** 10.7759/cureus.95285

**Published:** 2025-10-24

**Authors:** Shahad F AlTayash, Sara Almutairi, Abdulrahman A Khan, Wael A Alsakran, Layan Altawil, Osama Alsheikh

**Affiliations:** 1 Ophthalmology, King Khaled Eye Specialist Hospital, Riyadh, SAU; 2 Vitreoretinal Division, King Khaled Eye Specialist Hospital, Riyadh, SAU; 3 Oculoplastic and Orbit, King Khaled Eye Specialist Hospital, Riyadh, SAU

**Keywords:** chronic kidney disease, diabetes mellitus, disseminated intravascular coagulation, intravitreal bevacizumab, panretinal laser photocoagulation

## Abstract

A 42-year-old man with poorly controlled diabetes mellitus, hypertension, chronic kidney disease, and a diabetic foot received treatment with panretinal laser photocoagulation in his right eye and intravitreal injection of Bevacizumab in both eyes to manage proliferative diabetic retinopathy accompanied by diabetic macular edema in the right eye and complicated by vitreous hemorrhage in the left eye. He then presented on the same day with right eye pain, eyelid swelling, ecchymosis, and subconjunctival hemorrhage. Additionally, he showed right-sided signs suggestive of facial palsy. Laboratory tests revealed a significantly low platelet count. Orbital and brain imaging ruled out retrobulbar hemorrhage and ischemic insults. A referral was made to a tertiary hospital for further evaluation and treatment of suspected disseminated intravascular coagulation (DIC) by a multidisciplinary team. To the best of our knowledge, the development of DIC after ocular procedures has not been previously reported. We recommend careful management of patients with poorly controlled diabetes and kidney disease to prevent further systemic complications.

## Introduction

Disseminated intravascular coagulation (DIC) is an acquired syndrome characterized by impaired regulation of coagulation and inflammatory responses, leading to the activation of intravascular coagulation associated with microvascular damage [1,2]. It is commonly associated with critical conditions, including major trauma, sepsis, neoplasms, and obstetric complications [3]. In DIC, the creation of thrombin, induced by inflammation and vascular endothelial damage, contributes to the development of increased microthrombi, impairing organ perfusion and ultimately causing organ failure [4]. Concerning the ocular region, DIC has been associated with anterior and posterior segment hemorrhages accompanied by fibrin deposition [5,6]. However, no study has linked the development of DIC to it being caused by or following ocular interventions. Herein, we report a novel case of DIC in a patient with multiple systemic comorbidities, which directly developed after receiving combined treatment of retinal laser photocoagulation and intravitreal bevacizumab injection to address his proliferative diabetic retinopathy.

## Case presentation

**Figure 1:**
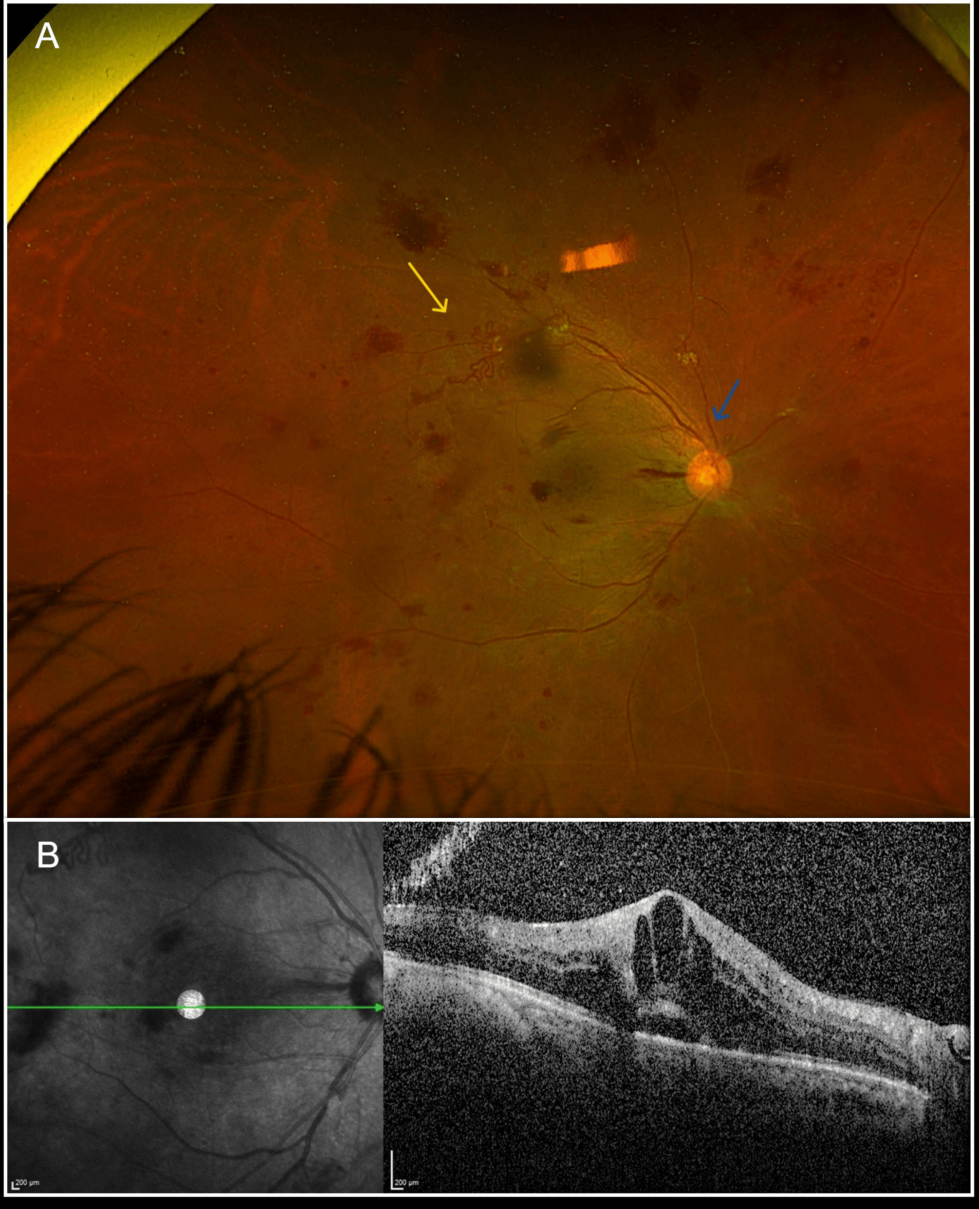
Color fundus of the right eye and macular optical coherence tomography images at presentation. (A) Wide-field fundus photo of the right eye showing neovascularization of the disc (blue arrow) and neovascularization elsewhere (yellow arrow). (B) Macular optical coherence tomography of the right eye showing a center involving cystoid macular edema.

A 42-year-old man, a current smoker, with a past medical history significant for poorly controlled type 2 diabetes mellitus complicated by prior diabetic foot infection, hypertension, and chronic kidney disease, presented to the emergency department complaining of sudden painless loss of vision in the left eye, which started three days before presentation. On examination, the patient had visual acuity of 20/200 in the right eye and hand motion in the left eye. Intraocular pressure was 16 mmHg in the right eye and 18 mmHg in the left eye. Extraocular motility was full in both eyes. Pupils were round, regular, and reactive with no afferent pupillary defect. Slit lamp examination revealed clear corneas, deep and quiet anterior chambers, normal irises, and nuclear sclerotic cataracts in both eyes. A dilated fundus exam (DFE) showed clear vitreous in the right eye with a flat retina and neovascularization of the disc (NVD) and neovascularization elsewhere (NVE) (Figure 1A). The left eye DFE demonstrated a hazy vitreous with a poor view of the retina, consistent with dense vitreous hemorrhage. Macular optical coherence tomography (OCT) revealed a center involving diabetic macular edema (DME) in the right eye (Figure 1B).

Macular OCT of the left eye could not be obtained as the retina could not be visualized. A B-scan ultrasound of the left eye was performed, which revealed an extremely dense vitreous opacity with dense organized posterior hyphema, and no retinal detachment was seen. The patient was diagnosed with active high-risk proliferative diabetic retinopathy (PDR) in the right eye and vitreous hemorrhage secondary to PDR in the left eye. The patient subsequently received panretinal laser photocoagulation (PRP) to the right eye and an intravitreal bevacizumab 1.25 mg injection to both eyes to treat DME in the right eye and to treat the vitreous hemorrhage in the left eye.

**Figure 2:**
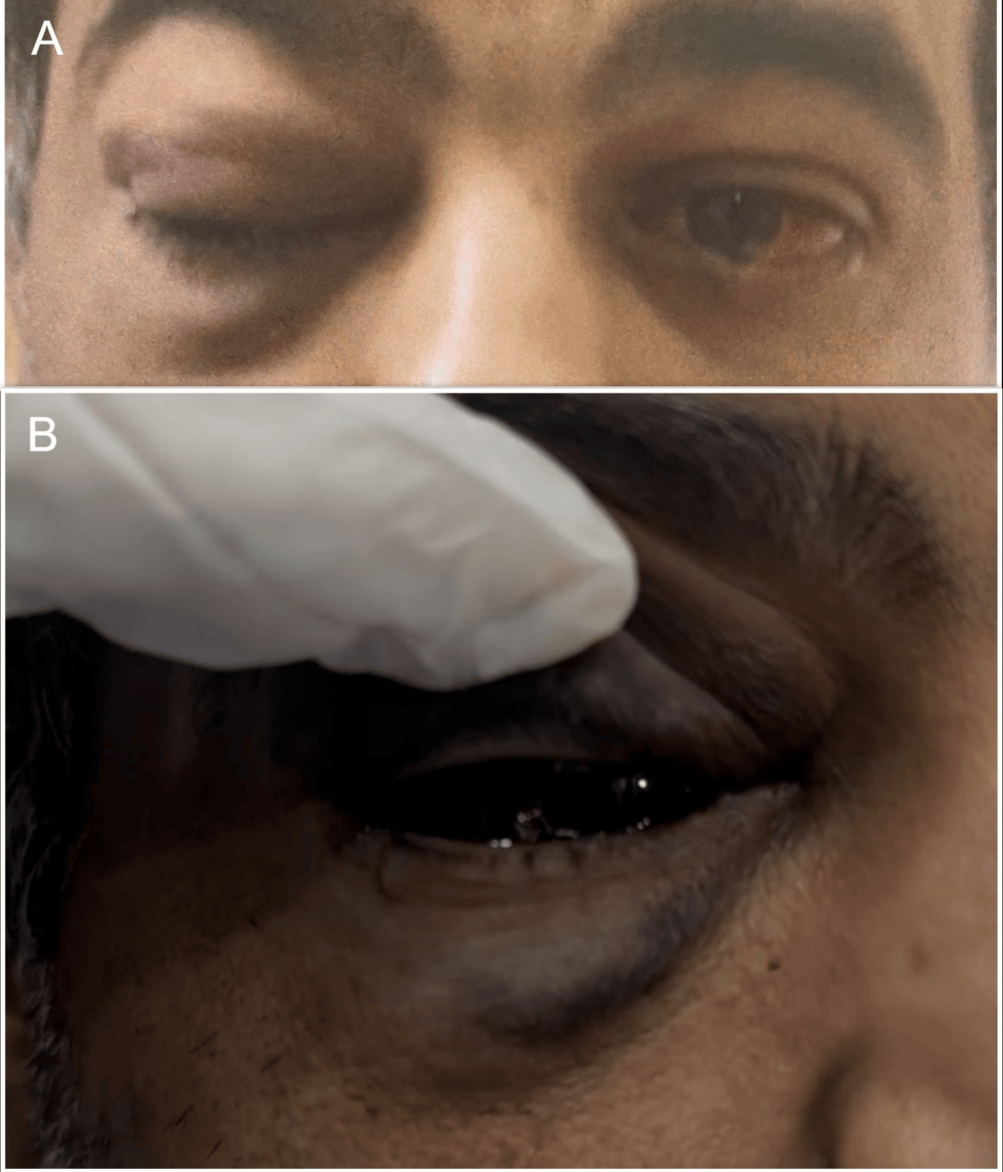
External images (A) External image of both eyes showing the right eye upper and lower lid swelling with ecchymosis. (B) External image of the right eye demonstrating diffuse subconjunctival hemorrhage.

The patient presented to the emergency department later on the same day, six hours after receiving the bevacizumab injection to both eyes, complaining of right upper and lower lid swelling, right eye redness, and right eye pain, associated with epistaxis. The patient was drowsy and not feeling well. His vital signs showed an elevated blood pressure of 185/98 mmHg. His Glasgow Coma Scale was 15/15. Upon examination, his visual acuity remained the same in both eyes. Intraocular pressure was 43 mmHg in the right eye and 30 mmHg in the left. The external exam revealed mild right upper and lower lid swelling with ecchymosis, subconjunctival hemorrhage, and no proptosis (Figure 2).

Extraocular muscle motility was full with no limitation. Facial asymmetry with a right-sided depressed mouth angle and loss of nasolabial fold, suggesting right facial nerve paresis, was noted. The remainder of the slit lamp and fundus exam was unchanged. Our differential diagnosis at this time included retrobulbar hemorrhage, carotid cavernous fistula, and orbital cellulitis with cavernous sinus thrombosis. A basic lab workup was performed demonstrating significantly low platelet levels, slightly prolonged prothrombin time (PT) and activated partial thromboplastin time (aPTT), as well as elevated creatinine and blood urea nitrogen. Table 1 summarizes the basic laboratory results.

**Table 1:** Laboratory work up at initial presentation

Labs	Value	Unit	Reference range
White blood cell (WBC)	9.6	10^9^/L	3.8-10.6
Platelets	14	10^9^/L	150-450
Prothrombin time (PT)	14.5	Seconds	10-13
Activated partial thromboplastin time (aPTT)	40.8	Seconds	25-35
International normalized ratio (INR)	1.08		0.9-1.2
Erythrocyte sedimentation rate (ESR)	75	mm/h	0-10
Creatinine	285	μmol/L	33-115
Blood urea nitrogen (BUN)	14.3	mmol/L	2.5-6.4

**Figure 3:**
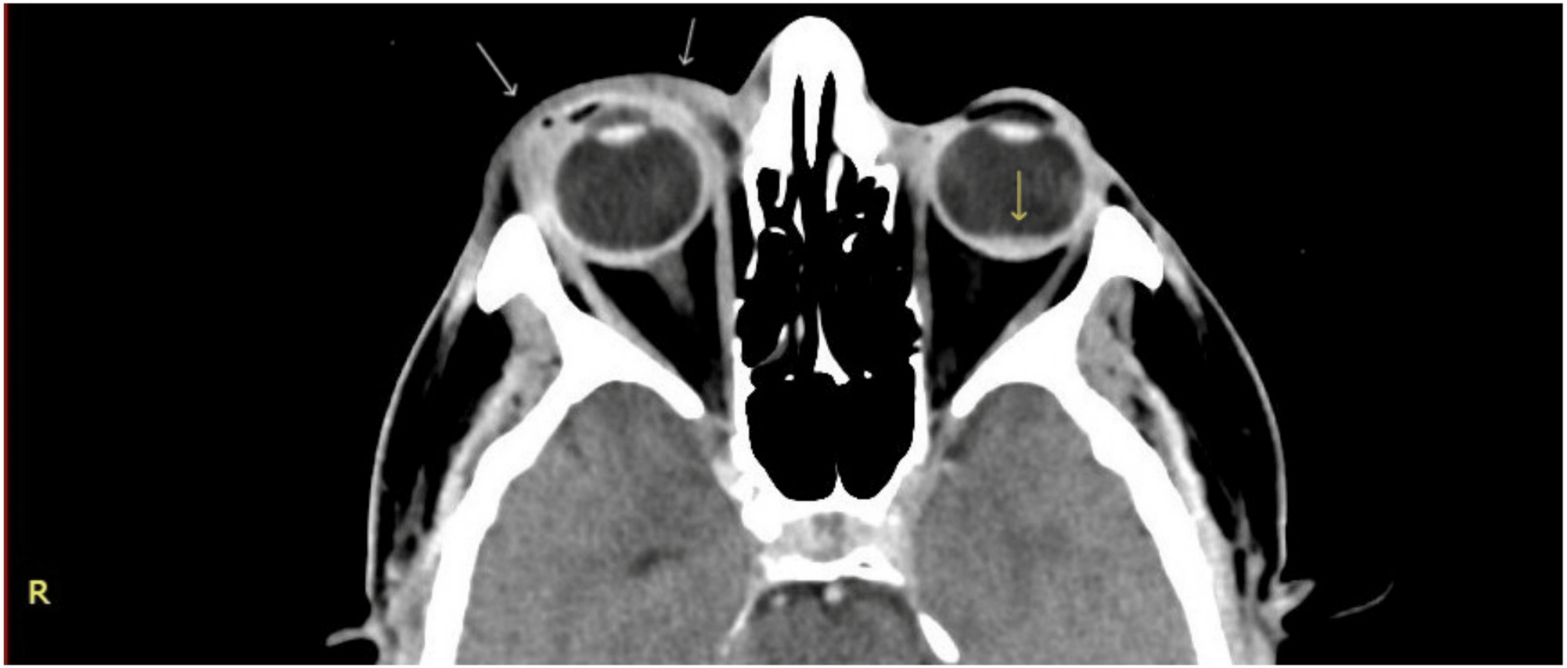
Axial view contrast enhanced computed tomography (CT) scan of the orbits Mild swelling and enhancement of the right eyelids, with mild related preseptal soft tissue swelling (white arrows) with no collection or post-septal extension. Left globe shows minimal high density fluid level in the vitreous (yellow arrow). No retrobulbar hemorrhage or lesions on both sides. No globe protrusion on both sides.

Computed tomography (CT) imaging of the brain and orbits, in addition to vascular imaging, revealed mild swelling and enhancement of the right eyelids and preseptal soft tissue with no radiographic signs of a fistula, hemorrhage, ischemia, or inflammatory process. Left globe showed minimal fluid levels consistent with vitreous hemorrhage (Figure 3). 

The patient’s intraocular pressure was reduced to 28 mmHg in the right eye and 20 mmHg in the left eye one hour after starting topical antiglaucoma drops in the emergency department. The patient was subsequently transferred to a medical facility for evaluation and management by a multidisciplinary team.

The general medical assessment involving the cardiovascular system, chest, and abdomen was unremarkable. Skin examination revealed no petechiae, purpura, or extensive bruising, except for the documented right foot scarred wound, which did not show any sign of current infection. A swab from his diabetic foot scarred wound was sent for culture and turned out negative for infection. Despite the patient's right facial nerve palsy, ischemic stroke was ruled out after a repeat CT brain imaging as well as dedicated vascular imaging. Given the constellation of his findings, including low platelet count, the acute presentation with bleeding tendency after the ocular interventions, and the presence of multiple risk factors in the context of his comorbidities, the diagnosis of disseminated intravascular coagulation (DIC) was made. Laboratory investigations, including blood cultures and tumor markers, turned out unremarkable, which excluded other risk factors for DIC. During his hospital stay, thrombocytopenia was treated by the hematology team with 12 units of platelet transfusion, as well as a single dose of human normal immunoglobulin 5%. After improvement, he was discharged in stable condition and started on oral prednisolone 1 mg/kg with quick tapering every three days.

**Figure 4:**
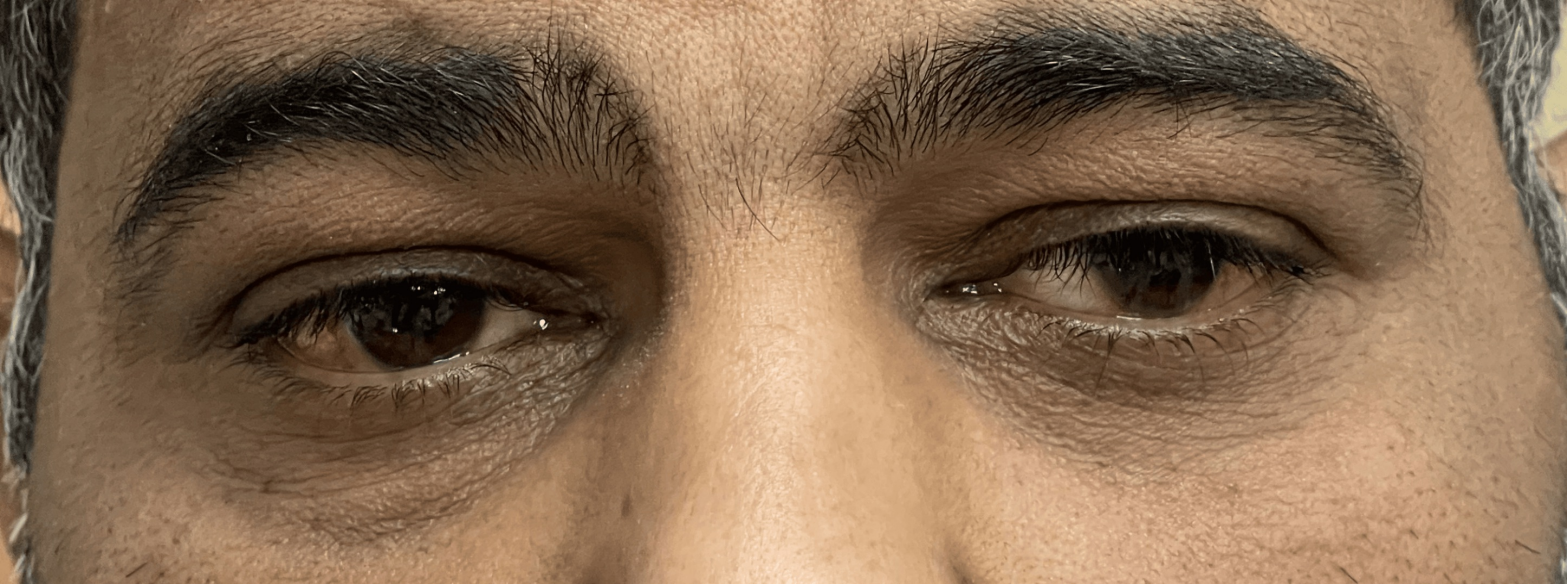
External image one month after initial presentation External image of both eyes showing resolution of lid swelling and ecchymosis.

The patient presented one month later to the retina clinic for follow-up. His latest laboratory investigations showed normalization of the platelet levels and an unremarkable coagulation profile. Upon examination, visual acuity was 20/200 in the right eye and light perception in the left eye. Intraocular pressure was 16 and 15 in the right and left eyes, respectively. His external examination revealed resolution of lid edema, and extraocular motility was normal with no limitation (Figure 4).

**Figure 5:**
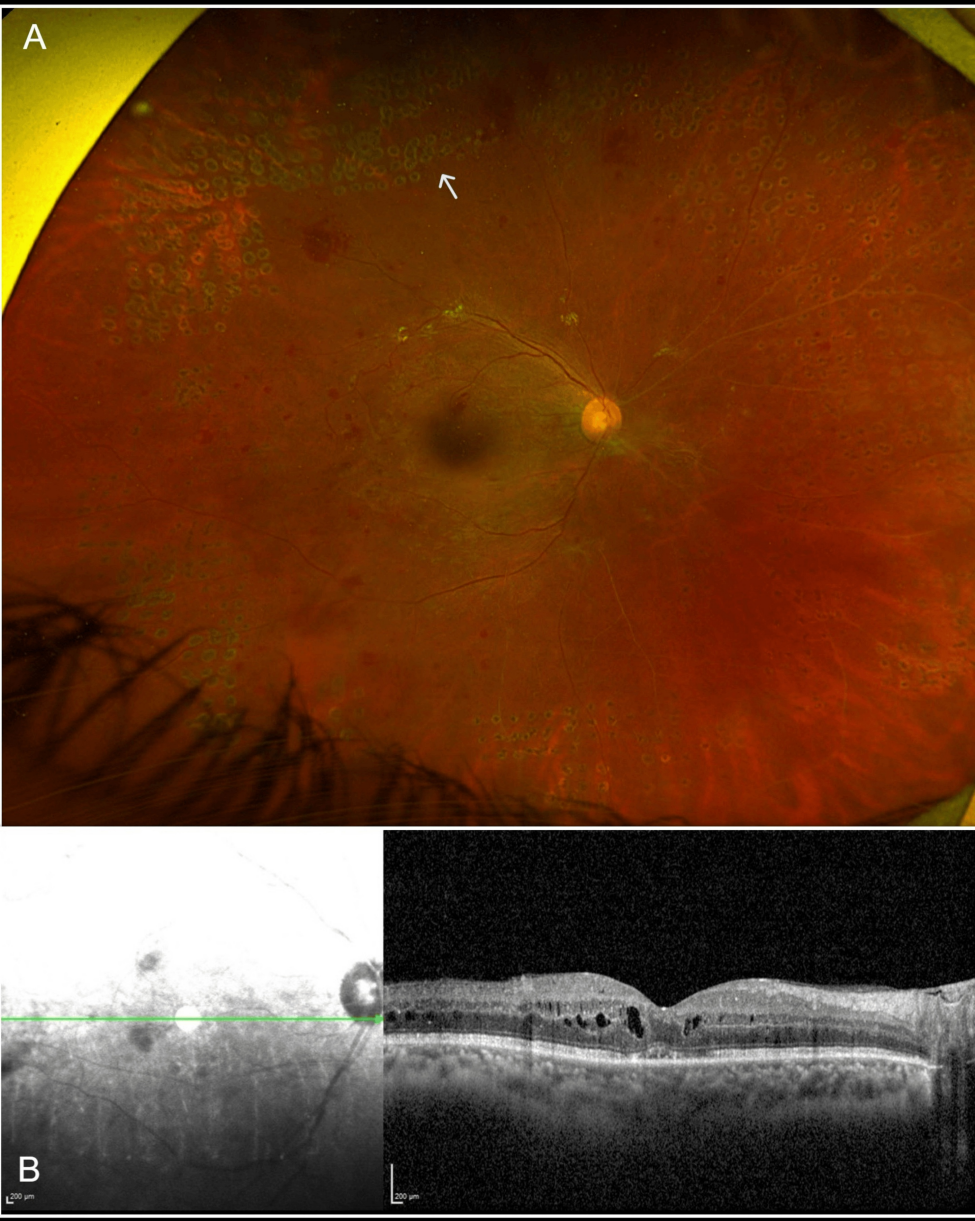
Color fundus and macular optical coherence tomography images one month after initial presentation (A) Wide-field fundus photo of the right eye one month after treatment with panretinal laser photocoagulation and intravitreal injection of bevacizumab, showing regression of neovascularization of the disc and elsewhere, and panretinal photocoagulation scars (white arrow). (B) Macular optical coherence tomography of the right eye one month after treatment showing improvement of diabetic macular edema.

Facial expressions were symmetric with no signs of facial nerve palsy. Slit lamp exam of the anterior segment was the same as the baseline exam and did not disclose any neovascularization of the iris in both eyes. DFE of the right eye shows regressing NVD and NVE, as well as scattered PRP scars (Figure 5A). The left eye DFE has not changed, with dense VH still obscuring further view. A repeat B-scan ultrasound of the left eye showed the same findings, with no retinal detachment observed. OCT of the right macula revealed significant improvement of DME (Figure 5B).

## Discussion

DIC is characterized by acquired activation of the coagulation system, resulting in microvascular thrombosis and potentially life-threatening bleeding owing to consumption of coagulation components and platelets [7]. The uninhibited coagulative and inflammatory response due to a major systemic morbidity is the pivotal point for the development of DIC [2,3]. Rare reports have documented DIC with concomitant ophthalmic abnormalities, as opposed to the relatively more usual relationship in other body regions [5,6,8]. Ocular involvement in DIC has been reported previously to affect the choroidal vessels, which may lead to choroidal hemorrhage or serous retinal detachment [9,10]. Lid edema, ecchymosis, hyphema, vitreous hemorrhage, and orbital hemorrhage, which can cause compartment syndrome, are the main reported ophthalmic signs associated with DIC [5,6,8]. Our patient resembled previously described cases in which he had lid edema and ecchymosis, which might be attributed to the nature of consumptive coagulopathy in the context of DIC. Given that our patient experienced a rise in intraocular pressure and limitation of ocular motility, orbital compartment syndrome was initially suspected but ruled out by imaging studies. Additionally, the existence of retinal vascular proliferation in the affected eye before our interventions strongly implies that the vitreous hemorrhage in the other eye is caused by advanced diabetic retinopathy rather than DIC.

DIC can be viewed as the last characteristic or consequence of a variety of systemic disorders. These mostly involve sepsis, malignancy, or following significant trauma or major surgical intervention [3,11]. However, our described case included several other coupled factors that would explain the development of DIC. We believe that the patient's kidney disease status is one of the major factors. Acute and chronic renal illness can impact the coagulation system and protease-dependent signaling [12]. Significant basal activation of blood coagulation and reduction of natural anticoagulants were reported in association with chronic kidney disease [13]. In addition, uremia and platelet dysfunction in the setting of severe renal impairment are a proven association that can predispose to a hypercoagulable state [14]. These pathological features of renal insufficiency have the potential to induce DIC.

On the other hand, decreased fibrinolysis and activation of prothrombotic coagulative factors are well-known to manifest in patients with diabetes mellitus. Consequently, diabetic patients are at a higher risk of DIC, particularly if associated with systemic comorbidities that may predispose them to hypercoagulation [15]. Diabetic foot-induced sepsis is one of the major complications in the context of uncontrolled diabetes mellitus. Since sepsis is a well-known cause of DIC, it was critical to exclude diabetic foot ulcers in our patient [11]. Our patient's plantar wound was clean and showed no signs of infection or inflammation. In addition, infection was ruled out following an unremarkable tissue sampling and laboratory tests. From our perspective, the existence of a diabetic foot wound, even if clinically clean and free of infection, may contribute to a procoagulant condition in vulnerable individuals. Diabetic neuropathy increases the risk of unrecognized recurrent microtrauma, which causes localized tissue hypoxia and poor wound healing. Such wounds are characterized by endothelial dysfunction, persistent low-grade oxidative stress, and dysregulated angiogenic signaling, which may add to the risk of DIC formation [16]. 

Iatrogenic and medication-induced etiologies of DIC are established risk factors. A significant variety of drugs have been linked to an elevated risk of DIC [17]. Bevacizumab is an anti-neoplastic medication that works primarily by inhibiting the receptors of vascular endothelial growth factor (VEGF), which limits the formation of new blood vessels and effectively disrupts a tumor's supply of oxygen and nutrients [18]. Because of its anti-angiogenic characteristics, intravitreal bevacizumab has been used effectively to treat proliferative diabetic retinopathy and macular edema [19]. Although extremely uncommon, systemic hypercoagulable vasculopathy has been documented after receiving intravitreal bevacizumab injections. The main reported findings were elevated blood pressure, variable cranial nerve palsy, myocardial infarction, ischemic stroke, and death [20-22]. Interestingly, our patient presented with an ipsilateral facial nerve palsy, which has not been previously reported in the setting of intravitreal bevacizumab injection, as opposed to oculomotor or abducens nerve palsy [21,22].

Systemic ablative procedures in the form of laser ablation and cryoablation have been linked with DIC development [23,24]. It was hypothesized that the processes involved are the release of tissue factors and cytokines due to tissue destruction, together with endothelial injury, which may activate coagulation and cause platelet consumption [23,24]. Panretinal photocoagulation (PRP) laser is the standard of care for managing proliferative diabetic retinopathy [25]. Although DIC has been reported after large-volume tissue ablation procedures, PRP and ocular cryotherapy have never been linked to DIC, most likely because of their confined injury region, limited systemic inflammatory response, and minor tissue damage. However, we believe that our patient acquired DIC because of a combination of vascular endothelial damage caused by his uncontrolled systemic comorbidities and iatrogenic pro-coagulative procedures, which might explain this unique relationship.

## Conclusions

DIC is extremely unusual to be associated with ocular or orbital signs, and it has never been shown to occur following ocular operations; yet a high level of suspicion is required to detect and treat such a case promptly. Intravitreal injections and laser photocoagulation are considered minor operations; however, they are not risk-free. Arranging for routine laboratory investigations before minor ocular interventions can be considered, especially for very vulnerable patients, to avoid such a rare complication and ensure safe procedures.
